# Catheter Ablation of Hemodynamically Unstable Ventricular Tachycardia in a Patient with Dextro - Transposition of the Great Arteries and Mustard's Repair

**Published:** 2011-07-03

**Authors:** Girish M Nair, Jeffrey S Healey, Elaine Gordon, Syamkumar Divakaramenon, Carlos A Morillo

**Affiliations:** McMaster University and Hamilton Health Sciences

**Keywords:** Catheter Ablation, Ventricular Tachycardia, D-Transposition of Great Arteries, Mustard's Repair, Defibrillator shocks

## Abstract

**Introduction:**

A patient with D-TGA and surgical repair (Mustard's procedure) presented with appropriate ICD shocks due to monomorphic ventricular tachycardia, refractory to antiarrhythmic medications.

**Methods and Results:**

The patient underwent an electrophysiological study and catheter ablation for the VT. Substrate and pace mapping techniques, with the help of an electroanatomical mapping system, was used to localize and ablate the tachycardia successfully.

**Conclusions:**

In patients with D-TGA and Mustard's repair, scar tissue resulting from VSD repair can act as a substrate for recurrent VT. Catheter ablation of VT is useful in management of VT that occurs despite antiarrhythmic therapy and/or when it is unstable.

## Background

Dextro- transposition of the great arteries (D-TGA) with palliative intra-atrial redirection surgery (Mustard or Senning procedures) has one of the highest sudden cardiac death (SCD) risks in patients with congenital heart disease. In patients with D-TGA high rates of appropriate Implantable Cardioverter- Defibrillator (ICD) shocks for ventricular tachycarrhythmias have been noted [1-3]. We report the case of a patient with D-TGA, and an ICD presenting with drug refractory ventricular tachycardia and frequent ICD shocks, who underwent catheter ablation.

## Methods and Results

Our patient is a 35 years old man, with D-TGA, ventricular septal defect (VSD), and mild sub-pulmonic stenosis (PS) at birth. He underwent atrial septostomy with pulmonary artery (PA) banding (Blalock-Hanlon) three months after birth. Following this he underwent intra-atrial baffle rerouting (Mustard's repair) with closure of the VSD at three years of age.

In 2003 he survived a cardiac arrest secondary to ventricular fibrillation (VF) and was left with residual anoxic brain injury resulting in severe short-term memory impairment. He received a single chamber transvenous ICD following this event. During the ensuing years he had four appropriate ICD therapies for monomorphic VT varying in cycle length (CL) between 320 and 350 ms.

The patient was started on beta-blockers and Amiodarone (300 mg maintenance dose) for prevention of VT. He continued to have ICD shocks for monomorphic VT while on antiarrhythmic therapy. After discussion with the patient and his family, an electrophysiological (EP) study and catheter ablation for VT was performed. Cardiac imaging prior to the procedure showed systemic ventricular dysfunction with a calculated ejection fraction ranging from 35-40% along with mild venous ventricular systolic dysfunction. The intra-atrial baffles were patent and the atrioventricular and ventriculo-arterial valves were normal.

The EP study was performed under intravenous general anesthesia. Vascular access was obtained from the groin (three right femoral venous sheaths and one arterial sheath) using the Seldinger technique. ICD therapies were turned off and the ICD was programmed to the VOO pacing mode at a rate of 80 bpm. The EP study was performed while the patient was on 300 mg of Amiodarone and 5 mg of Bisoprolol a day.

**Figure 1:**
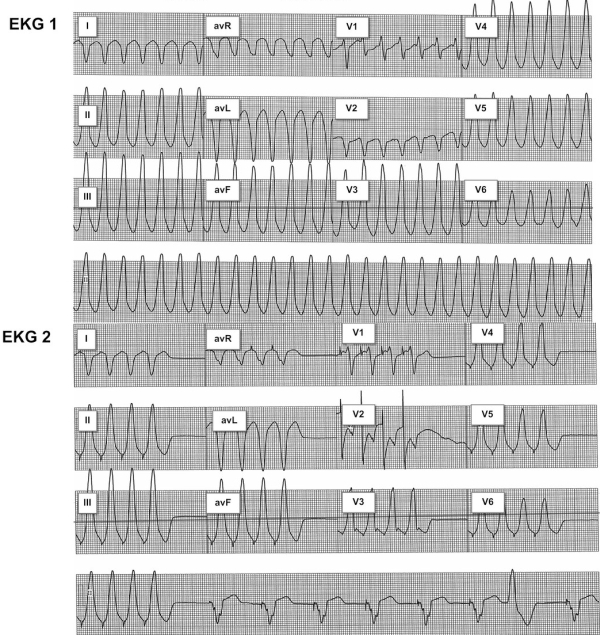
This figure shows 12 lead EKGs obtained during the EP study. Image 1 (top) shows the sustained monomorphic VT induced at the beginning of the study. Image 2 (bottom) shows the EKG with the best pace match obtained during pacemapping.

A sustained monomorphic VT with a CL of 350 ms was induced during programmed electrical stimulation (Figure 1). The patient was not able to tolerate the VT hemodynamically for more than 40 seconds. The tachycardia was terminated using an external, biphasic DC shock of 300 Joules. The 12 lead EKG during the VT showed an rS pattern in V1 with precordial transition in lead V3 and positive qRs complexes in leads II, III and aVF. Q waves were present in leads I, aVL and aVR. Based on these EKG findings we provisionally localized the VT to the septal region of the outflow tract of the systemic ventricle (anatomic RV in the sub aortic position).

A substrate map of the systemic ventricle was created using a non-fluoroscopic electroanatomical mapping system (CARTO, Biosense Webster Inc.) during ventricular pacing at a rate of 80 bpm (the patient had advanced AV conduction system disease and was pacing dependent). A Navistar thermocool catheter (3.5mm-tip saline-irrigated catheter; Biosense-Webster, Inc., Diamond Bar, CA) was used for electroanatomical mapping. Intravenous unfractionated heparin was used to keep the activated clotting time (ACT) > 240 seconds for the duration of the procedure.

**Figure 2:**
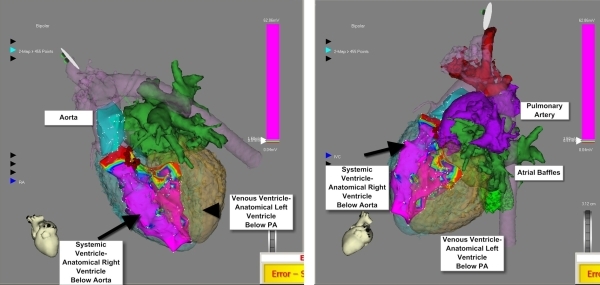
This image shows electroanatomical maps of the systemic ventricle and the ascending aorta integrated with the contrast enhanced CT image of the patient's heart. The image shows ablation lesions and the scarred interventricular septum of the heart.

Bipolar contact electrograms were recorded and used to create a three-dimensional map of the chamber, based on voltage amplitude. After creating an electroanatomical map of the ascending Aorta and the systemic ventricle we performed image integration (Figure 2). Areas with voltages greater than 1.5 mV were considered 'healthy' and those with voltages less than 0.5 mV were considered 'scarred'. Areas with voltages between 1.5 and 0.5 mV were considered 'unhealthy'. A discrete scarred region in the ventricular septal region of the systemic ventricle, just below the Aorta, was observed. This area correlated with the site of VSD patch repair. The scar occupied one third of the basal interventricular septum (Figure 2).

**Figure 3:**
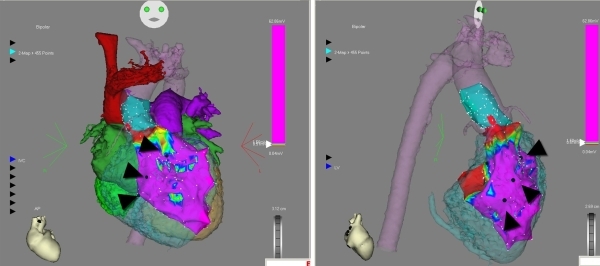
This image shows electroanatomical maps of the systemic ventricle and the ascending aorta integrated with the contrast enhanced CT image of the patient's heart. The image shows sites used for pacemapping during the EP study.

**Figure 4:**
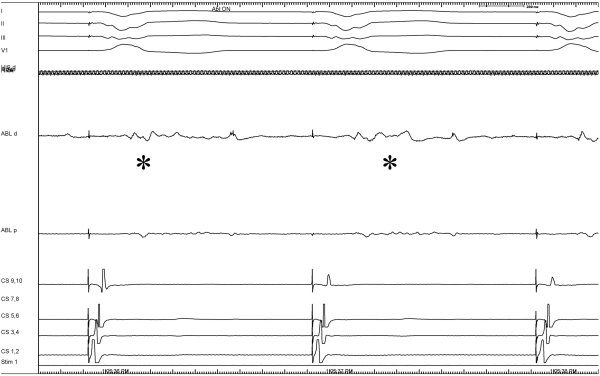
This image shows local bipolar electrograms from the ablation catheter. The indicates signals from the distal ablation catheter. Fragmented, low voltage signals (0.5-1.5mV) were recorded from the edge of the scar in the interventricular septum in the outflow tract of the systemic ventricle below the aortic valve. Ablations were delivered in this region rendering the VT non-inducible.

We then performed pace mapping at a CL of 450 ms at different sites in the systemic ventricle till we obtained a 12/12 match with the induced tachycardia. The site of 12/12-pace match was at the edge of the scar in the interventricular septum, in the systemic ventricle just below the Aortic coronary cusp (Figure 3). Local bipolar electrograms at this site and all along the scar edge showed fragmented low voltage electrograms (Figure 4). Linear radiofrequency ablation lesions (power of 40 W for a maximum duration of 180 s at a time) were delivered from the exit site toward the center of the substrate and along the scar border zone perpendicular to the first line. Each line was composed of multiple sequential lesions placed ~5 mm apart. All lesions were made at the 1.0 - 1.5 mV border.  Ablations were continued until the local electrogram was reduced to < 25% (of the amplitude at the start of the ablation), converted to a qs complex or split [4,5].

After RF ablation, attempts to reinduce VT, including programmed electrical and burst pacing from the systemic and venous ventricle were undertaken. No ventricular arrhythmias were induced. We deemed this a satisfactory endpoint, as the VT was easily inducible prior to catheter ablation. The patient withstood the procedure without any acute complications. The patient is being followed up in the ICD clinic and has not had any further ventricular arrhythmias during the past ten months. Amiodarone has been discontinued and the patient continues to be on Bisoprolol.

## Discussion

Our patient was selected for catheter ablation of VT as he was having multiple appropriate ICD shocks while on maximally tolerated antiarrhythmic medications. The patient had a single- inducible; scar related, monomorphic VT that was not hemodynamically tolerated. The CL of the VT was identical to VTs recorded by the patient's ICD.  Activation mapping and entrainment maneuvers could not be performed because of hemodynamic instability. Therefore, substrate and pacemapping to localize the origin of the VT were performed.

Unlike entrainment mapping, which is performed while the patient is in VT, one cannot be certain about whether a particular site is within a critical portion of a VT circuit. The best pacemap site provides a general idea of where the VT exits allowing the operator to deliver targeted lesions around critical areas of the VT circuit. This is a probabilistic approach to VT ablation with limitations, but it has the advantage of being performed in sinus rhythm - thereby allowing the operator to target even hemodynamically unstable VTs [4,5]. We had an acceptable acute endpoint of non-inducibility of VT after the catheter ablation. However, the patient was loaded on Amiodarone and it is possible that VT could have been induced had the patient not been on antiarrhythmic medication.

Zeppenfeld and colleagues have presented insights from VT ablation in patients with tetralogy of Fallot (TOF) and intracardiac repair. They identified isthmuses around the region of patch annuloplasty and ventricular septal defect repair in the venous ventricle thought to be responsible for genesis of ventricular arrhythmias. Catheter ablation transecting anatomical isthmuses around scars along with entrainment pace mapping and substrate ablation was successful in preventing arrhythmia recurrence in these patients. In our case the clinical VT originated in the systemic ventricle and was not hemodynamically well tolerated. This prevented us from performing entrainment mapping and delineating the arrhythmia circuit. Therefore, we resorted to pace mapping to localize the exit site of the VT and used voltage based substrate ablation in and around the scar. We were not able to identify a clear anatomical isthmus at this site (with the Aortic or mitral annulus) and therefore did not perform transecting linear isthmus ablation as described by Zeppenfeld [4].

Advances in imaging and mapping technologies have made ablation of arrhythmias in patients with congenital heart disease safe and effective. However, difficulties in obtaining vascular access to cardiac chambers and the potential for major complications such as cardiac perforation, intra-atrial baffle tears and damage to cardiac valves should be kept in mind while selecting patients for catheter ablation.

## Conclusions

In patients with D-TGA and Mustard's repair scar tissue resulting from VSD repair can act as a substrate for recurrent VT. Catheter ablation of VT is useful in management of VT that occurs despite antiarrhythmic therapy and/or when it is unstable.
